# Histopathological, morphological, and molecular characterization of fish-borne zoonotic parasite *Eustrongylides Excisus* infecting Northern pike (*Esox lucius*) in Iran

**DOI:** 10.1186/s12917-024-04146-0

**Published:** 2024-07-04

**Authors:** Hooman Rahmati-Holasoo, Mohammad Azizzadeh, Hosseinali Ebrahimzadeh Mousavi, Sara Shokrpoor, Zahra Ziafati Kafi, Amin Marandi

**Affiliations:** 1 Department of Aquatic Animal Health, Faculty of Veterinary Medicine, University of Tehran, Tehran, Iran; 2 Department of Pathobiology, Faculty of Veterinary Medicine, University of Tehran, Tehran, Iran; 3 Department of Microbiology and Immunology, Faculty of Veterinary Medicine, University of Tehran, Tehran, Iran

**Keywords:** *Eustrongylides excisus*, Granulomatous myositis, Northern pike, Freshwater ecosystem, Iran

## Abstract

*Eustrongylides excisus* is a fish-borne zoonotic parasite known to infect various fish species, including Northern pike (*Esox Lucius*). This nematode, belonging to the family Dioctophymatidae, has a complex life cycle involving multiple hosts. This study aimed to investigate the occurrence of *Eustrongylides* nematodes in Northern pike (*E. Lucius*) collected from Mijran Dam (Ramsar, Iran). Between June and October 2023, an investigation was conducted on Northern pike from Mijran Dam in Ramsar, Iran, following reports of reddish parasites in their muscle tissues. Sixty fish were examined at the University of Tehran, revealing live parasites in the muscles, which were then analyzed microscopically and preserved for a multidisciplinary study. The skeletal muscle tissues of 85% (51/60) of fish specimens were infected by grossly visible larvae which were microscopically identified as *Eustrongylides* spp. In histopathological examination, the lesion was composed of encapsulated parasitic granulomatous myositis. Microscopically, the cystic parasitic granulomas compressed the adjacent muscle fibers, leading to their atrophy and Zenker’s necrosis. Moreover, epithelioid macrophages, giant cells and mononuclear inflammatory cells were present around the larvae and between the muscle fibers. Finally, a molecular analysis by examining the ITS gene region, revealed that they belong to the species *E. excisus*. Eustrongylidiasis in northern Iran necessitates further research into the biology, epidemiology, and control of *Eustrongylides* nematodes, focusing on various hosts. This study is the first to comprehensively characterize *E. excisus* in Northern pike in Ramsar, Iran, raising concerns about possible zoonotic transmission.

## Introduction

Aquaculture is a rapidly expanding field globally, mostly focused on producing fish for human consumption [1–3]. The northern pike (*Esox Lucius* Linnaeus, 1758) is a freshwater food fish that has been belonged to the family Esocidae [4]. This fish, is known as a predatory fish species that is found in freshwater lakes and rivers in the northern hemisphere. The northern pike holds significant economic value as a game fish in both North America and Eurasia, owing to its substantial size and extensive geographical range [5, 6]. Although this fish species originally distributed in the freshwater, it is capable of surviving in slightly brackish environments [7, 8]. Commercial fishing operations target the Northern pike (*E. lucius*), which is extremely valuable for human consumption and feeds on a diverse range of food items, including invertebrates and fish [9, 10]. The International Union for Conservation of Nature (IUCN) has mentioned the Northern pike (*E. lucius*) as least concerned in the list of threatened species [11].

Various fish species are supposedly experiencing an increase in the prevalence and incidence of emerging diseases on a global scale. The development of control and management strategies for these diseases requires an initial understanding of their etiology and the host, as well as potential consequences [12, 13]. Parasitic infestations pose a significant challenge to fish farming, thereby exerting a substantial impact on the global aquaculture industry [14, 15]. The mortality and economic losses caused by parasitic pathogens infecting teleost fish are widely recognized in both the aquaculture sector and in wild fish populations [16–18]. In addition, fish species that are profitable might harbour parasite larvae of food hygienic importance [19]. The genus *Eustrongylides* Jagerskiold, 1909, belongs to the family Dioctophymatidae, involving red and grossly visible nematodes that exhibit a cosmopolitan distribution across Northern and Southern America, Europe, and Asia [20–24] and display complex life cycle that encompasses both primary and secondary intermediate hosts, along with definitive hosts [25, 26]. The nematoda genus *Eustrongylides* has numerous described species, but according to Measures [27] and Honcharov et al. [28], only three of these species-*Eustrongylides tubifex*, *E. excisus*, and *E. ignotus*-are valid. *Eustrongylides* spp. have an indirect heteroxenous life cycle, and adult nematodes inhabit the mucosa of the oesophagus, proventriculus, or intestine of piscivore birds such as Ciconiiformes, Anseriformes, Gaviiformes, and Pelecaniformes [29, 30]. Oligochaetes, particularly ubificidae and Lumbriculidae, serve as the initial intermediate hosts after the eggs are released into the aquatic environment through feces [27, 31]. Moreover, planktivorous, benthivorous, and pelagic fish, as well as amphibians and/or reptiles, are known as the second intermediate hosts. According to Coyner et al. [32] and Menconi et al. [33], Eustrongylid nematodes have been documented in 17 different orders of fish globally. In addition, predatory fish such as pike (*Esox* spp.) and pike perch (*Sander lucioperca*) can play the role of paratenic hosts, while humans may be accidental hosts [27, 34, 35]. The most important species of the genus *Eustrongylides*, which are potentially zoonotic nematodes that infect freshwater fish, is *E. excisus* [36]. *Eustrongylides excisus* has been reported in several fish species, such as pike-perch *Sander lucioperca* [24, 37, 38], big-scale sand smelt *Atherina boyeri* [25, 39, 40], European perch (*Perca fluviatilis*) [41], great cormorant (*Phalacrocorax carbo*) [41], black bullhead *Ameiurus melas* [25], wels catfish *Silurus glanis* [25], pumpkinseed *Lepomis gibbosus* [25], largemouth black bass *Micropterus salmoides* [25], and thin lip grey mullet *Chelon ramada* [25]. Thus, the present study provided comprehensive histopathological, morphological, and molecular characterization of the fish-borne zoonotic parasite *Eustrongylides excisus* infecting Northern pike (*E. lucius*) in Iran.

## Materials and methods

### Fish sampling and laboratory examination

Following suspicious reports from local fishermen regarding the presence of several apparent reddish parasites in the muscle tissues of Northern pike (*E. lucius*) caught from Mijran Dam, located in Ramsar, Iran, a thorough investigation was conducted between June and October 2023. A total of 60 specimens of Northern pike measuring between 30 and 50 cm in length, were randomly collected from Mijran Dam, and carefully placed in plastic bags filled with water. The bags were equipped with oxygen supply in order to safeguard the welfare of the fish during transportation. The Northern pikes were then transported to the aquatic animal lab, Faculty of Veterinary Medicine at the University of Tehran, located in Tehran, Iran. Wet smears of external organs were prepared and subsequently observed using light microscopy (E600, Nikon).

### Parasitological examination

Prior to the examination, the fish were subjected to euthanasia by administering an overdose of PI222 (Pars Imen Daru, Iran) (10 ml/10 lit), which containing Eugenol, Carvacrol, and Eugenol acetate as its major active ingredients. Following the euthanasia procedure, the ventral surface of each fish was opened longitudinally, axial muscles were incised (five cuts per specimen) and several grossly visible reddish parasites in the muscle tissues of the fish specimens were collected. In order to identify visible parasites, all of these procedures were carried out under controlled lighting. Subsequently, the removed parasites underwent detailed microscopic examination using a trinocular stereomicroscope microscopy (SZ60, Olympus) and light microscopy (BX41, Olympus and E600, Nikon). Following the carmine-based staining and bright-field (BF), dark-field (DF), and differential interference contrast (DIC) imaging using an GT 12 microscope digital camera (Tucsen, China) (Fig. 2a-d), parasites were preserved in a 70% ethanol solution for further molecular analysis. In addition, 16 parasites were removed from the fish and preserved in a fish-free, source- and tap water-containing environment at 25 °C in order to investigate their potential stability in an aquatic ecosystem devoid of fish.

**Fig. 1:**
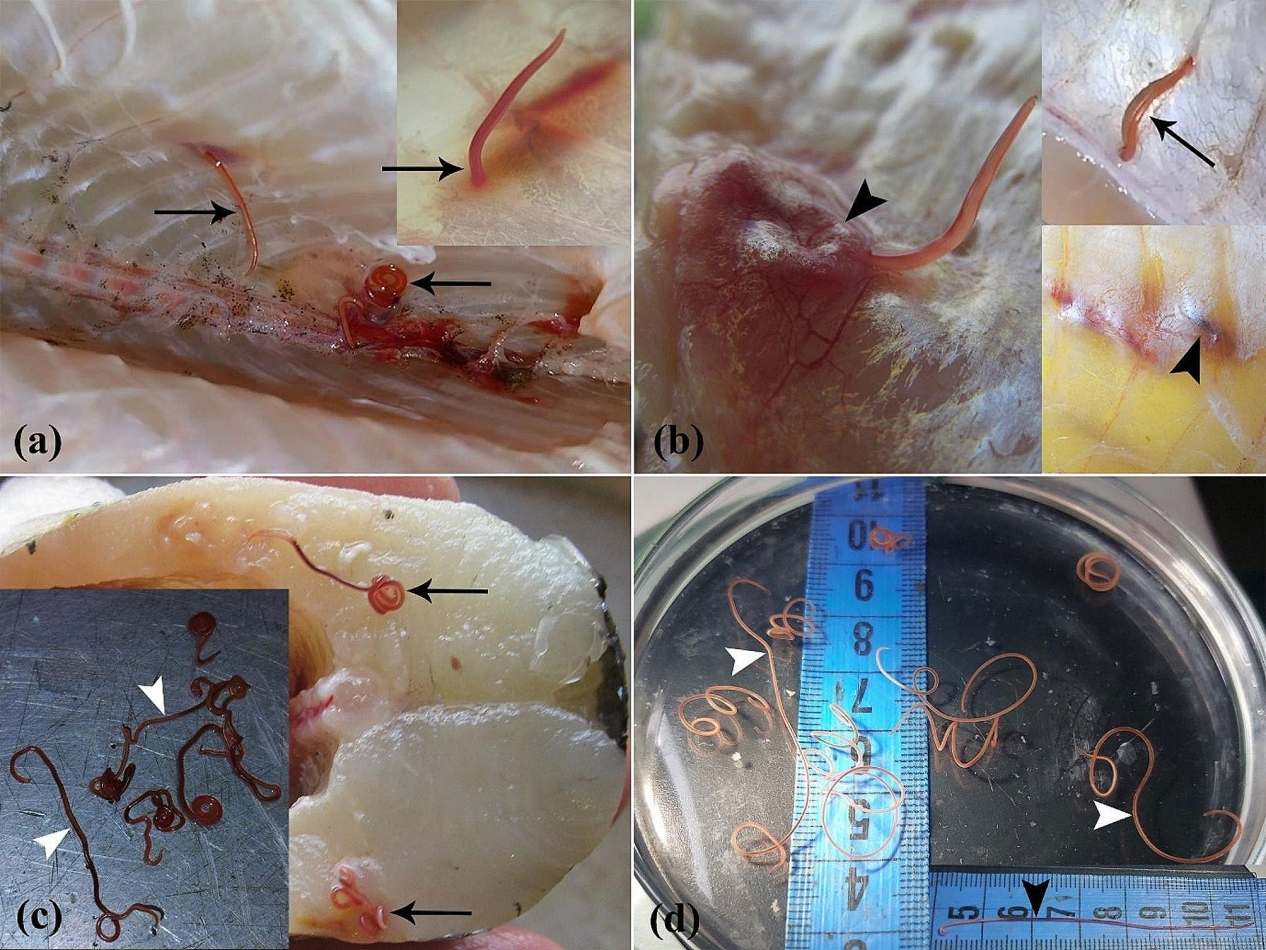
(**a**-**d**) Macroscopic aspect of Northern pike (*E. lucius*) invasion with *E. excisus*. (**a**) Nematodes are found within the supra-axial muscles and in close proximity to the vertebral column (arrows). (**b**) Tissue reaction to the presence of parasites in the muscle tissues (arrowheads) and the nematode in motion (arrow). (**c**) Nematodes are found in supra-axial muscles (arrows), and dark red alive nematodes are removed from the fish (arrowheads). (**d**) Significant discoloration of multiple live nematodes 9 days after isolation from the fish (white arrowheads) compared to the nematode 2 days after isolation from the fish (black arrowhead)

### Histopathological analysis

Parasite-containing skeletal muscle tissue samples were fixed at 10% neutral buffered formalin (NBF). Following this, the tissues underwent dehydration procedures through a sequence of ethanol solutions and were subsequently prepared for embedding in paraffin using a paraffin tissue processor and paraffin dispenser. Following that, sections were sliced at a thickness of 4 μm. These sections were then subjected to staining procedures using hematoxylin-eosin (H&E) and Masson’s trichrome (MT), followed by examination under a light microscope (E600, Nikon). In addition, representative images were taken utilizing a microscope camera (GT 12, Tucsen, Mosaic 3.0 software) and scaled using Axiovision 4.8 software, and the sections of the tissues were scanned by the TrueChrome Metrics microscope camera (Tucsen, Mosaic 3.0 software).

### DNA extraction and polymerase chain reaction (PCR)

The samples were crushed and homogenized with 400 μl of the homogenization buffer of the KPG DNA extraction kit. 200 μl of the homogenized samples were transferred for DNA extraction, and total DNA was extracted using the same kit according to the guidelines provided by the manufacturer, Karmania Pars Gene (KPG), Iran. In addition, the extracted DNA was eluted in 45 μl of RNAase-free sterile water and stored at -80 °C until the following step. The PCR reaction system consisted of a 25 μl mixture containing 12 μl of the ready-to-use Amplicon^®^ master mix, 8 μl ddH2O, and 1 μl forward and reverse primers. For this reaction, a primer pair 18SF (TTGGATGATTCGGTGAGGT) and 28SR (AACCGCTTAGTAATATGCT) was used to amplify a 995 bp fragment of ITS rDNA. The PCR reaction was performed according to the following procedure: initial denaturation at 95 °C for 5 min, then denaturation at 95 °C for 1 min, annealing at 50 °C for 1 min, and extension at 72 °C for 2 min. The PCR reaction was carried out for 39 cycles, followed by a final extension at 72 °C for 5 min. To analyze the PCR products, 5 μl of each PCR product was electrophoresed in a 1.5% agarose gel. The agarose gel was stained with ethidium bromide and visualized with a UV transilluminator. Sequencing of positive samples was subsequently initiated.

### Sequencing, bioinformatics, and phylogenetic analysis

Positive PCR products were sequenced individually by Codon genetic group (Tehran, Iran) using the Sanger sequencing method. The quality of the sequences was first evaluated with NCBI BLASTN tool on the National Center for Biotechnology Information (NCBI) website (https://blast.ncbi.nlm.nih.gov/) and then with Finch TV software. Furthermore, alignment and phylogenetic analysis were performed using MEGA 11.0 software [42]. The phylogenetic tree was constructed based on the maximum likelihood (ML) method and the general reversible time model. The reliability of the tree was estimated using the bootstrap method with 1000 replicates.

## Results

### Clinical examination

The postmortem examinations under sterile conditions revealed grossly visible live parasites (ranging between 5 and 7 cm in length, and between 1 and 12 per fish) in the skeletal muscle tissues of 85% (51/60) of fish specimens. Notably, the majority of these curled parasites were found within the supra-axial muscles and in close proximity to the vertebral column (Fig. 1a-c). In addition, after preserving the parasites in a fish-free, source- and tap water-containing environment at 25 °C to investigate their potential stability in an aquatic ecosystem devoid of fish, it has been revealed that despite becoming paler and less mobile over time, the parasites managed to survive the 10-day period in host-free water (Fig. 1d).

**Fig. 2:**
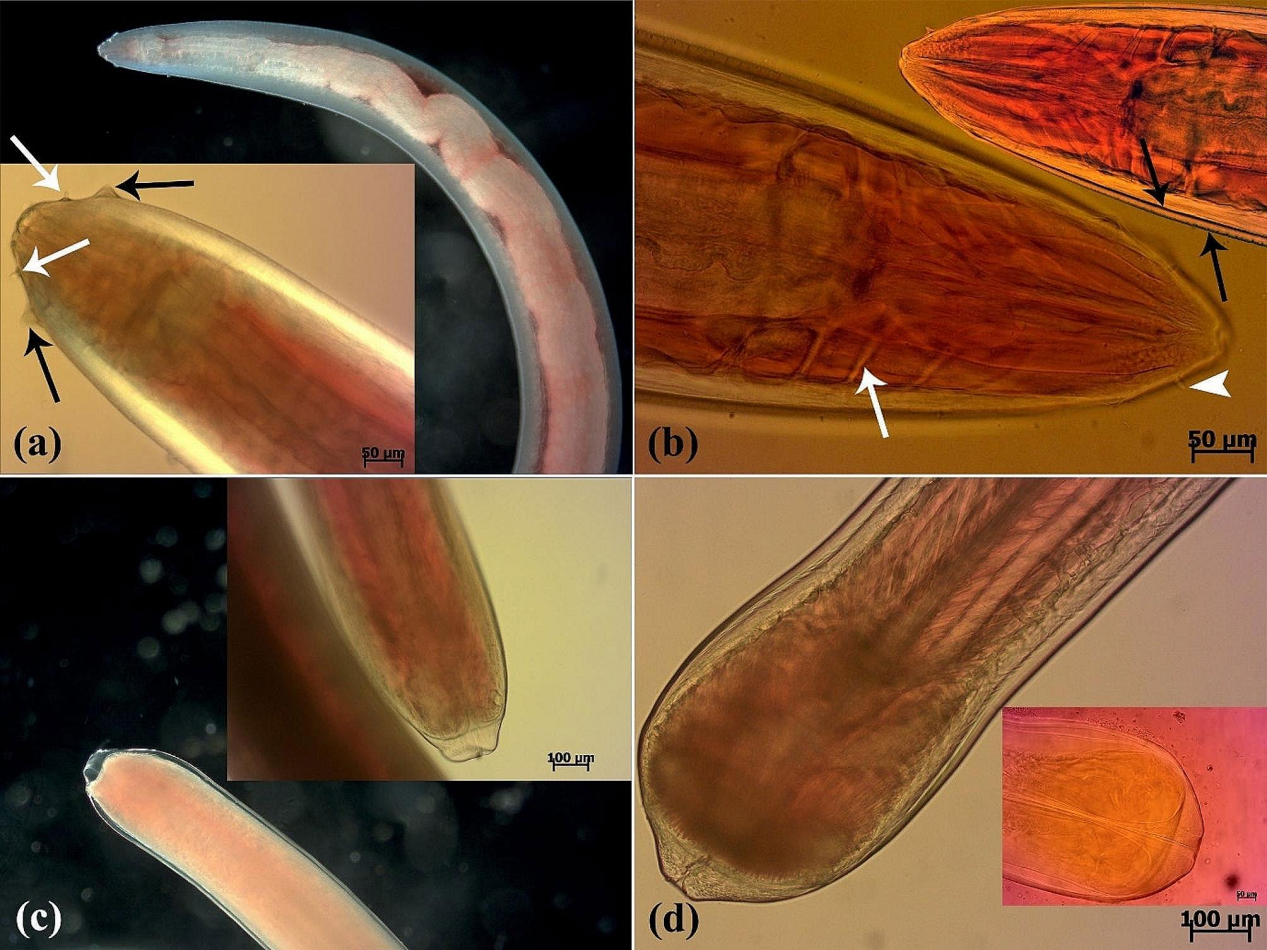
(**a**-**d**) Microscopic aspect of *Eustrongyloides excisus* isolated from Northern pike (*E. lucius*). (**a**) Dark-field and DIC images of the anterior region of a live nematode. Two rows of labial papillae (both inner (white arrows) and outer (black arrows)) are observed (**b**) A light microscope image of the anterior extremity of an Azocarmine-stained nematode displaying neural rings (white arrow) and inner papillae (arrowhead). Two cuticle layers are observed (black arrows). (**c**) Dark-field and DIC images of the posterior region of a live female nematode. (**d**) A light microscope image of the posterior extremity of a live male nematode and an Azocarmine-stained nematode

### Parasitological identification

Following a comprehensive examination of the parasites using a trinocular stereomicroscope microscopy (SZ60, Olympus) and light microscopy (BX41, Olympus and E600, Nikon), carmine-based staining, and bright-field, dark-field, and differential interference contrast imaging, it was revealed that the body of the reddish nematodes widens towards the middle and narrows towards the ends. Moreover, the head end did not appear swollen, and the cuticle lacked spines. However, a deep ventral cleft with a row of cuticular projections was present on the caudal sucker at the caudal end of the adult males and the transverse outline of the parasite became more prominent towards the ends. Furthermore, the nematodes were identified as belonging to the taxonomic genus *Eustrongylides* based on the presence of a small oral cavity surrounded by 12 cephalic papillae of similar size, two concentric rings, and genital primordia (Fig. 2a-d).

### Histopathological analysis

**Fig. 3:**
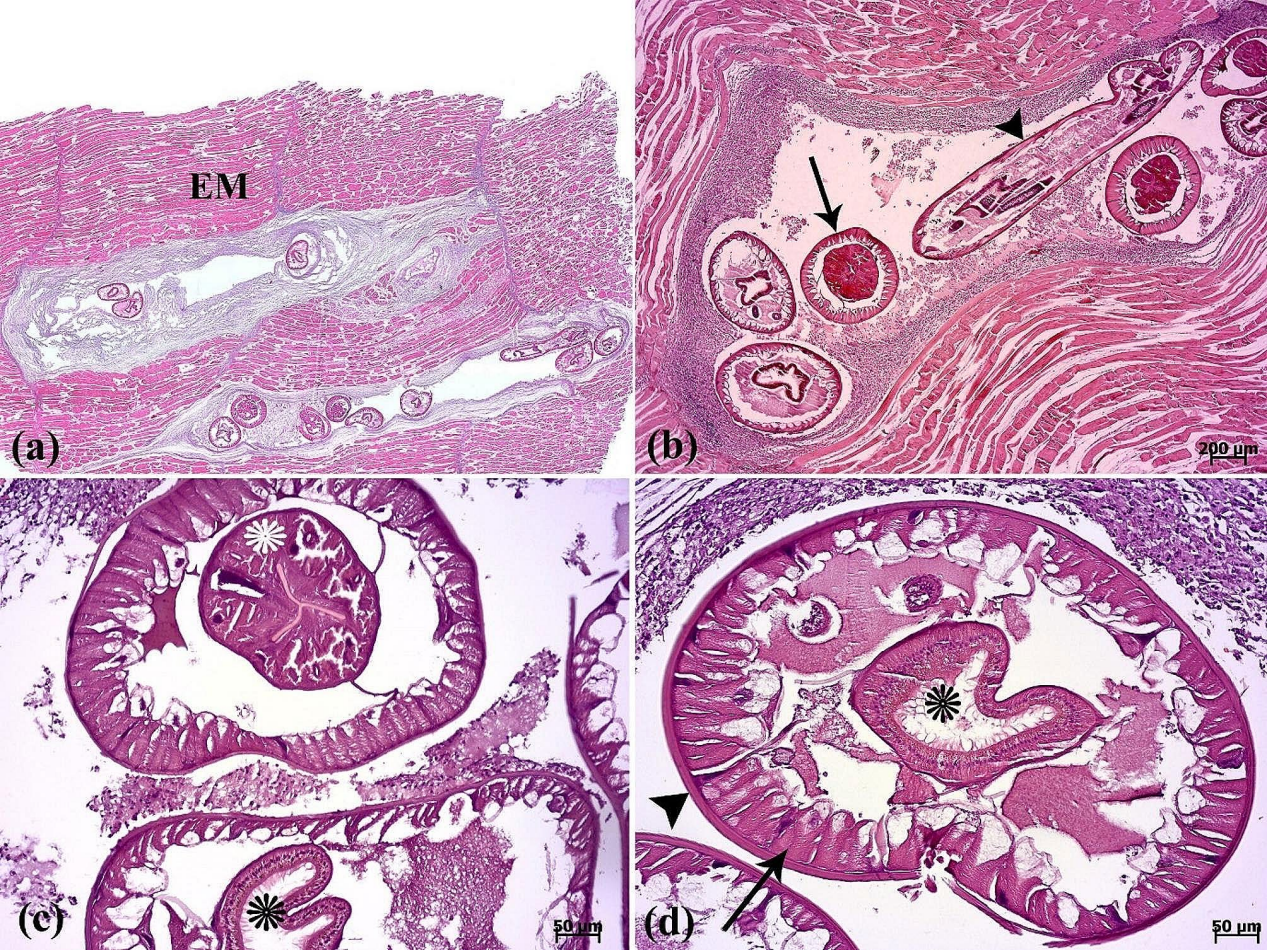
(**a**) The scanned image depicts a section of muscular tissue, revealing presence of parasites between the epaxial muscle fibers (EM). The nematodes have replaced large portions of the epaxial skeletal muscle. (**b**) Longitudinal (arrowhead) and transverse (arrow) sections of the parasite. (**c**-**d**) The cuticle (arrowhead), muscle fiber (arrow), esophagus (white asterisk), and intestine (black asterisks) in the transverse section of the parasite are seen

**Fig. 4:**
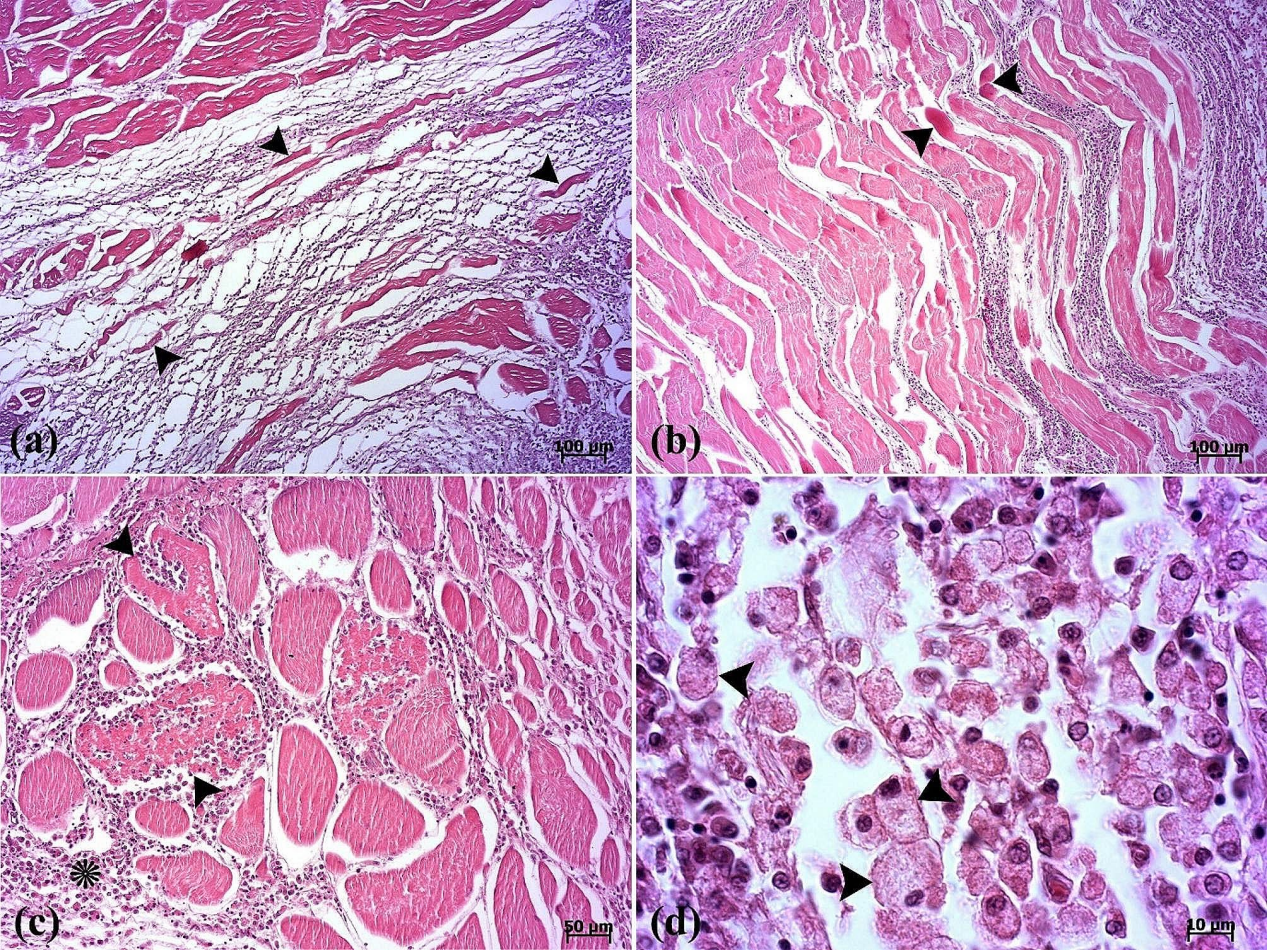
(**a**) Atrophy of muscle fibers (arrowheads) is seen. (**b**) Segmental necrosis (arrowheads). (**c**) Inflammatory cell infiltrations into the inter- (asterisk) and intramuscular (arrowheads) fibers are seen. (**d**) Note the presence of epithelioid macrophages (arrowheads)

**Fig. 5:**
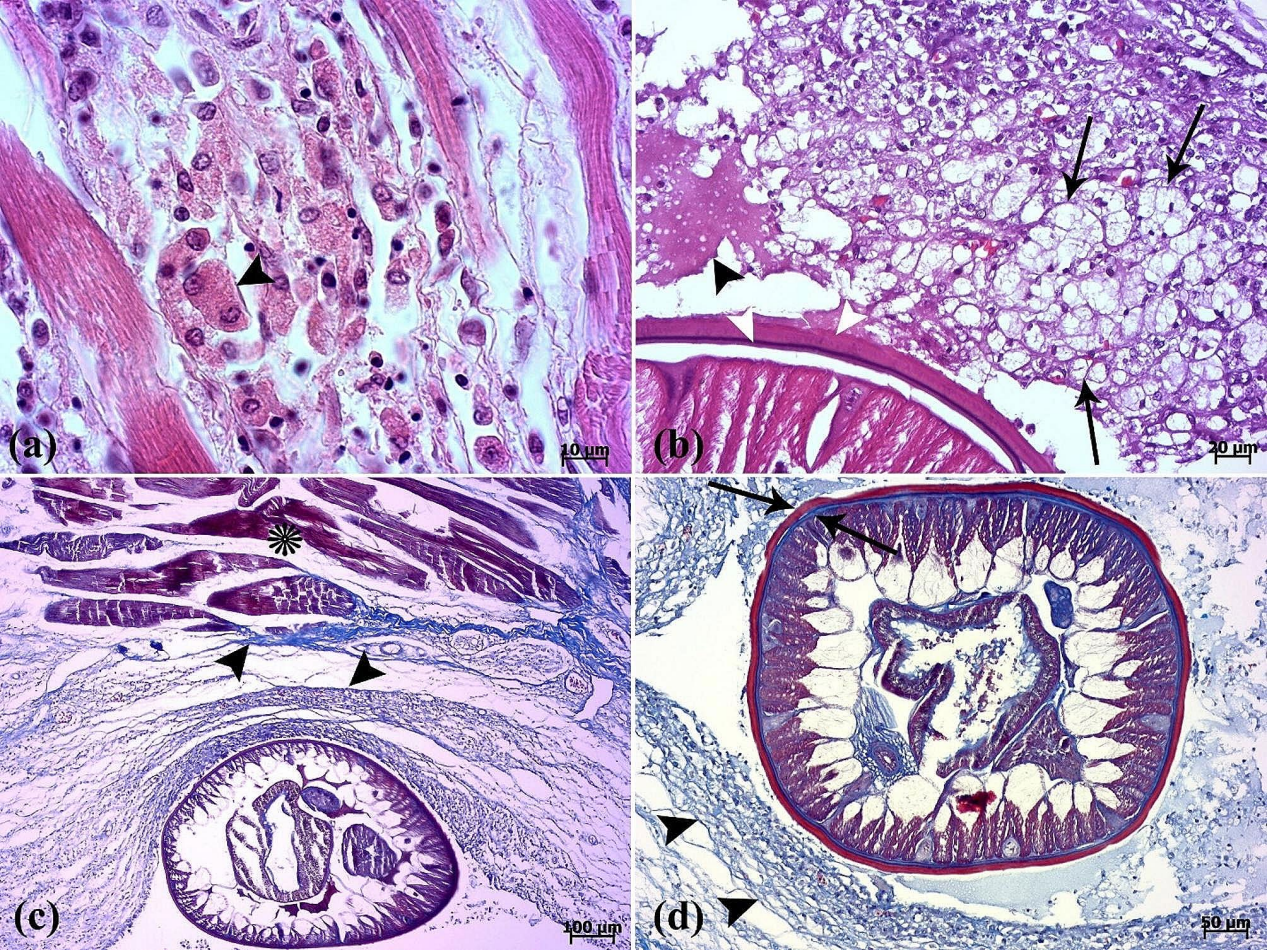
(**a**) A binucleated cell (arrowhead) is seen between muscle fibers. (**b**) Vacuolated cells (arrows) and eosinophilic material (arrowhead) in the lumen of the cyst are present. Two cuticle layers are observed (white arrowheads). (**c**-**d**) Masson’s trichrome is positive for collagenous fibers (arrowheads) surrounding the parasite. Muscle fibers (asterisk). External cuticle layer in red color and internal cuticle layer in blue color are seen (arrows)

The histopathological examination revealed that the lesion was composed of encapsulated parasitic granulomatous myositis, with longitudinal and transverse sections of the larvae were present in the muscle tissue (Fig. 3a-d). In the transverse sections of encapsulated larvae, the cuticle, longitudinal muscles, and digestive tract of the nematoda were distinguishable (Fig. 3c & d). Microscopically, the cystic parasitic granulomas compressed the adjacent muscle fibers, leading to their atrophy and Zenker’s necrosis (Fig. 4a & b). The larvae were surrounded by a capsule and exhibited infiltrations of inflammatory cells. Epithelioid macrophages, giant cells and mononuclear inflammatory cells (including lymphocytes and plasma cells) were diffused around the larvae and between the muscle fibers (Fig. 4c, d, & 5a). In addition, vacuolated cells, necrotic cells, and eosinophilic material were observed in the lumen of cysts (Fig. 5b). The cysts of the larval nematode consisted of a loose fibrous wall with fibroblasts, fibrocytes, and collagen bundles. Masson’s trichrome (MT) demonstrated the positive and blue staining of collagen in the cyst wall (Fig. 5c-d).

### Molecular and phylogenetic analysis

**Fig. 6:**
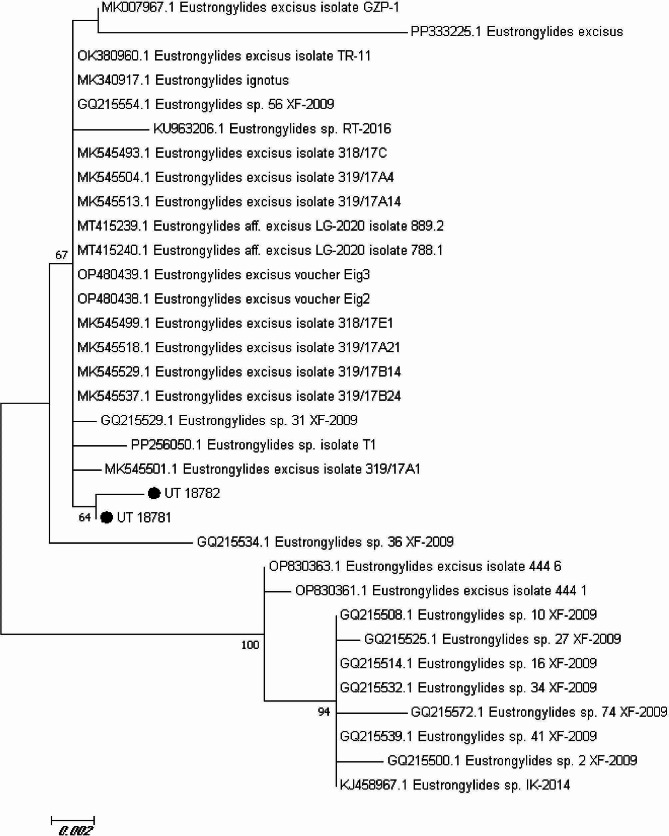
Molecular phylogenetic analysis based on the nucleotide sequences of the ITS rDNA gene was performed using the maximum likelihood method and the general time reversible model. The tree was generated for comparison of two isolates in the present study and some previously submitted to NCBI GeneBank

The BLAST results showed that both isolates in the present study (UT-18,781 and UT-18,782) have a high similarity (99%) with *Estrongylides* species. The phylogenetic analysis also revealed that both sequences belong to *Estrongylides* species. Moreover, comparison of the present isolates with some previously submitted isolates showed a high similarity between UT-18,781 and OP480438 from Turkey (99.89%), MK545529, MK545518, MT415240 and OK380960 from Italy (99.87%), MK007967, another isolate from Turkey (99.78%) and GQ215532 from China (97.69%). These results also showed a high similarity for the other isolate UT-18,782 with OP480438 (99.67%), MK545529, MK545518 and OK380960 (99.62%), MT415240 (99.61%), MK007967 (99.56%), GQ215532 (97.43) (Fig. 6; Table 1).

**Table 1:** Nucleotide sequence variation for the Eustrongylids isolated in the present study and 8 previously submitted isolates in the NCBI GeneBank based on the ITS rDNA Gene

		1	2	3	4	5	6	7	8	9	10
1	UT_18781										
2	UT_18782	99.78									
3	MK545529.1_Eustrongylides_excisus_isolate_319/17B14	99.87	99.62								
4	MK545518.1_Eustrongylides_excisus_isolate_319/17A21	99.87	99.62	100.00							
5	MK545499.1_Eustrongylides_excisus_isolate_318/17E1	99.87	99.62	100.00	100.00						
6	OP480438.1_Eustrongylides_excisus_voucher_Eig2	99.89	99.67	100.00	100.00	100.00					
7	MT415240.1_Eustrongylides_aff._excisus_LG-2020_isolate_788.1	99.87	99.61	100.00	100.00	100.00	100.00				
8	OK380960.1_Eustrongylides_excisus_isolate_TR-11	99.87	99.62	100.00	100.00	100.00	100.00	100.00			
9	MK007967.1_Eustrongylides_excisus_isolate_GZP-1	99.78	99.56	100.00	100.00	99.87	99.89	100.00	99.87		
10	GQ215532.1_Eustrongylides_sp._34_XF-2009	97.69	97.43	97.56	97.56	97.48	97.81	97.53	97.57	97.69	

## Discussion

The parasitic infestations are one of the most concerning problems affecting the aquaculture industry [43, 44]. The majority of individual fish in both wild and cultivated populations are infected with parasitic pathogens, which may affect the function, growth, reproduction, and survival of the hosts [43–45]. The *Eustrongylides* species are gaining significant interest owing to their extensive geographic distribution and remarkable potential for transmission and pathogenicity [40, 46]. Various host-related factors, including life stage, fish species and biological characteristics, as well as the abundance of intermediate and final hosts, may be associated with the incidence and prevalence of parasitic infections caused by *Eustrongylides* nematodes in Northern pikes in Mijran Dam. The reported associations between the occurrence and intensity of parasitism on one side, and fish species and life stage on the other side can be attributed to variations in feeding behavior [26, 35]. Early stages of the fish are more susceptible to Eustrongylidosis as they primarily consume zooplankton and benthic invertebrates (i.e., oligochaetes, which serve as the initial intermediate hosts of *E. excisus*). Whereas adults have a preference for consuming macroinvertebrates [47]. Furthermore, piscivorous fish species exhibit a higher susceptibility to infection compared to non-piscivorous fish species [26, 35]. The presence of favorable environmental conditions (e.g., nesting and habitation of fish-eating birds as the final hosts for *E. excisus*) is essential in facilitating the growth of oligochaete populations, particularly in locations where there is a high prevalence of *E. excisus* in fish. Furthermore, the diversity of infected species can be elucidated by the feeding behavior of fish, as predatory fish have the potential to consume multiple infected prey, thereby exacerbating the severity of parasite infection [32, 35].

Eustrongylides nematodes exhibit complex and indirect life cycles that involve aquatic oligochaetes as the initial intermediate hosts, followed by fish, amphibians, and/or reptiles as second intermediate or paratenic hosts, and finally piscivorous birds as the definitive hosts [48]. Eustrongylides nematodes begin their developmental process when eggs are laid on an aquatic oligochaete. The larvae then penetrate the intestinal wall and enter the body cavity of the host [28]. After the process of double shedding and parasitic invasion for a duration of 82–85 days, the fish become the secondary intermediate host and acquire infection through the ingestion of oligochaetes that are infected [49]. According to Eberhard and Ruiz-Tiben [50], the larvae experience multiple shedding and subsequently developed to the 3rd (L3) and 4th (L4) development stages of development as they infest along the fish’s body. According to Cole [49], the larval stages exhibit a duration that surpasses one year. Piscivorous avian species or mammalian organisms, which serve as definitive hosts, acquire infections through the consumption of infected fish. In addition, transmission of dioctophymids may be significantly influenced by paratenic hosts, such as frog and fish species that are commonly infected [50]. The parasite is located within the gastric wall of fish and undergoes maturation over a span of 10–15 days [28]. The eggs possess the ability to maintain vital functions for a span of 2-2.5 years, during which they undergo a maturation process lasting 19–21 days in an external environment prior to reaching the invasive stage. The ideal conditions for the eggs are water that is saturated with organic matter and a temperature range of 20 to 30 °C [49].

Parasites that impact freshwater fish species have the potential to cause severe diseases in human populations [19, 25]. *Eustrongylides* species do not typically infect humans. Nevertheless, there are instances where they may assume the role of hosts through the ingestion of parasitized raw or poorly cooked fish and fish products, resulting in the manifestation of intense abdominal discomfort, gastritis, and intestinal perforations [26, 28, 35, 40, 50–54]. The prevalence of Eustrongylides infection in various fish species globally is primarily observed in fish that are consumed by humans [28]. Parasitism by *Eustrongylides* spp. in larger edible fish typically does not cause noticeable clinical symptoms such as abdominal distension or muscle bulge. However, in certain cases, it may be linked to abnormal swimming behaviors. The transmission of parasitic infection through the consumption of infected but apparently healthy fish is particularly significant because the disease can be transmitted between humans and animals, which is known as zoonosis [26, 28]. Due to medication residues, treatment should be applied cautiously to fish that humans consume. The larvae of *Eustrongylides* spp., on the other hand, can be found anywhere other than the intestine, rendering anti-helminthic treatment ineffective. Therefore, it is necessary to eliminate the entire colony if any specimens are found to be infected. Biosecurity measures involve avoiding areas with piscivorous birds, which serve as the final host, and regularly sanitizing ponds to eliminate oligochates, which are invertebrate intermediate hosts [55].

DIC light microscopy confirmed that the morphological features of *Eustrongyloides* larvae described herein were consistent with the previous descriptions of *Eustrongyloides excisus* reported by Bjelić-Čabrilo et al. [37], Çolak [39], Pekmezci and Bolukbas [24], Guardone et al. [40], Rusconi et al. [41], Öztürk and Öztürk [38], and Castiglione et al. [25]. The morphological characteristics, including a deep ventral cleft with a row of cuticular projections on the caudal sucker at the caudal end of the adult male as well as the shape and size of the labial papillae observed in this study, served as the most distinctive features that differentiate *E. excisus* from resembled species (e.g., *E. tubifex* and *E. ignotus*) [22, 24, 27].

The current capacity to identify *Eustrongylides* species through sequencing and phylogenetic analysis is restricted by the absence of deposited sequences of *E. tubifex*, *E. mergorum*, and *E. ignotus* derived from morphologically identified adult parasite specimens [56]. This study revealed that the sequence of UT-18,781 sample was completely identical to OP480438 from Turkey (99.89%), MK545529, MK545518, MT415240 and OK380960 from Italy (99.87%), MK007967, another isolate from Turkey (99.78%) and GQ215532 from China (97.69%). Moreover, results showed a high similarity for the other isolate UT-18,782 with OP480438 (99.67%), MK545529, MK545518 and OK380960 (99.62%), MT415240 (99.61%), MK007967 (99.56%) and GQ215532 (97.43%). While our phylogenetic analysis provides support for the hypothesis that *E. excisus* is the sole species inhabiting the entire Mijran Dam of Ramsar, additional research is required to definitively establish this.

The use of a control strategy aimed at interrupting the evolutionary life cycle of *Eustrongylides* nematodes through the elimination of the intermediate hosts may be regarded as one of the most potentially effective approaches to management. The heightened vigilance of health care providers and understanding, along with advancements in diagnostic tools, have led to a marked decline in the underdiagnosis of parasitic infections in fish and the subsequent identification of a large number of previously unrecognized cases, which likely contributes to the global rise in parasitic infections. The rising popularity of raw fish consumption complicates efforts to monitor and control parasites in fish at the consumer level. Supporting control efforts will be the implementation of more personal and regulatory actions, as well as food-safety measures such as the inspection of both local and imported fish and fish products. However, the enormous quantity of fish traded around the world makes it difficult to apply the desirable practice of detecting and eradicating these parasites in fish [57].

## Conclusions

While eustrongylidiasis has been verified in the northern region of Iran, additional parasitological investigations are required to elucidate the numerous aspects of the biology, epidemiology, and control of *Eustrongylides* nematodes in Iran. These investigations should focus on intermediate hosts such as oligochaetes and fish, as well as paratenic hosts like amphibians and reptiles and final hosts such as piscivorous birds. While it has not been definitively established that the species *E. excisus* is responsible for human cases, it is important to consider the possibility of zoonotic transmission until more information is gathered regarding the species involved in human cases and their geographical distribution. To the best of our knowledge, this study represents the first comprehensive histopathological, morphological, and molecular characterization of the fish-borne zoonotic parasite *Eustrongylides excisus* infecting Northern pike (*E. lucius*) in Ramsar, Iran.

## Data Availability

No datasets were generated or analysed during the current study.
